# Anticoagulant treatment at a specialized outpatient anticoagulant therapy unit, a descriptive study

**DOI:** 10.1186/1477-9560-3-20

**Published:** 2005-12-08

**Authors:** Kim Ekblom, Johan Hultdin, Bo Carlberg, Tage Strand

**Affiliations:** 1 Department of Medical Biosciences, Clinical Chemistry, Umeå University Hospital, 901 85 Umeå, Sweden; 2 Department of Public Health and Clinical Medicine, Medicine, Umeå University Hospital, 901 85 Umeå, Sweden

## Abstract

**Background:**

The indications for continuous oral anticoagulant treatment, the target interval and the procedures for withdrawing treatment have changed in the last 10 years.

**Methods:**

Patients on continuous oral anticoagulant treatment at the Outpatient Anticoagulant Clinic at Umeå University Hospital in 2002 were included in a descriptive study (n = 900). 263 of those had a mechanical heart valve prosthesis. Only patient records for patients with other indications than mechanical heart valve prosthesis were examined. 582 of those records were found. In the 55 remaining patients some clinical information was retrieved from the computerised warfarin dosage database. These latter, more unsure clinical data, are presented separately. Anticoagulant treatment was discontinued if lack of proper indication or presence of too high risk for hemorrhagic complications were found.

**Results:**

The prevalence of continuous oral anticoagulant treatment in the uptake area was 0.65%. The most common target interval was INR 2.1–3.0, but patients with a mechanical heart valve prosthesis were often treated more aggressively, i.e. with a higher INR target interval. Of the patients on continuous treatment, 26.6% of the INR values were outside 2.0–3.0. The most common reasons for oral anticoagulant treatment were atrial fibrillation or mechanical heart valve prosthesis, in contrast to earlier findings in studies of our population in 1987 and 1990. We found 90 patients (10.0%) without proper indication for oral anticoagulant treatment or too high risk, and their treatment was discontinued.

**Conclusion:**

In patients on oral anticoagulant therapy, re-evaluation of indications and risks resulted in a substantial number of treatment withdrawals. There have been major changes in treatment indications during the last decade, possibly due to rapid development of knowledge in the field of thrombosis risk factors. Treatment should be re-considered once a year.

## Background

Treatment with warfarin or other coumarin derivatives is an established method of secondary prevention after venous or arterial thromboembolic events, as well as for primary prevention. In 1997 the prevalence of oral anticoagulant treatment in Sweden was 0.8% [1]. That figure is comparable with those in Denmark, were the prevalence was 0.663% in 1997 and 0.784% in 1999 [2]. In 2002 19,823 kDDD (thousand defined daily doses) of Waran^® ^2.5 mg tablets (sodium warfarin) were sold in Sweden. In 2003 the number rose to 21,387 kDDD. In 1998 Apekumarol^® ^(dicoumarol) was withdrawn from the Swedish market. Marcoumar^® ^(phenprocoumon) can be prescribed on licence when warfarin cannot be used [3]. Nearly all patients now receive sodium warfarin in Sweden.

Oral anticoagulant treatment is a potentially dangerous medication. According to a study in Mölndal, Sweden, 4.5 per 100 patient years resulted in a serious bleeding complication leading to hospitalisation or persisting sequele. 0.5 per 100 patient years resulted in a patient dying of a bleeding complication [4]. Because of the possibility of serious, and sometimes lethal, complications it is necessary to ensure that each patient has a valid indication for continuous anticoagulant treatment. Every patient should be re-evaluated yearly according to the Swedish National Board of Health and Welfare.

In 1987–1990 a study compared the quality of oral anticoagulant treatment in the primary health care and in the Department of Internal Medicine at Umeå University hospital [5]. In 1990, 290 patients were treated in primary health care and 175 via the Department of Internal Medicine. Nowadays most patients in Umeå and the surrounding municipalities are treated via the specialized Outpatient Anticoagulant Clinic within the Department of Internal Medicine at Umeå University Hospital. Only a few patients are managed through primary health care centres.

The aims of this study were to describe patients on oral anticoagulant therapy in our outpatient clinic, to compare the results with a previous investigation, and re-evaluate indications and risks of complications in these patients.

## Methods

The cohort consisted of all patients receiving continuous oral anticoagulant therapy with either warfarin or coumarin derivatives on October 15, 2002, registered at the Outpatient Anticoagulant Clinic at the University Hospital of Umeå. It is the only hospital in the reception area. A small number of patients treated by general practitioners (n = 57) were not included in the study. In most cases monitoring by general practitioners is due to long distances in our catchment area. Due to the same reason, it may be estimated that the number of patients treated by other hospitals is negligible. During the time of the study, only one patient living in our area used a self-monitoring device, and he was supervised by the Outpatient Anticoagulant Clinic, and thus is included in our study.

All patients in the register with a treatment time defined as "indefinite" were considered to be on continuous oral anticoagulant therapy. The patient records from relevant clinics were examined. Personal data, start date of anticoagulant treatment, and all thromboembolic events in the patient's history that came to our knowledge were recorded. If previously unknown events were found in personal communication with the patient, these events were also recorded. For all patients without mechanical heart valve prosthesis, the reason for the ongoing anticoagulant treatment was recorded.

Mechanical valve prosthesis, dilated cardiomyopathia, or venous thrombosis, in combination with one of the following coagulopathies, were defined as absolute indications for continuous warfarin treatment: antithrombin deficiency, protein C deficiency, protein S deficiency, homozygote for F V Leiden (FV 1691 AA), homozygote for the prothrombin mutation (FII 20210 AA), heterozygote for both APC-resistance (FV 1691 GA) and prothrombin mutation (FII 20210 GA). If no problems with the treatment were registered by the nurses at the Outpatient Anticoagulant Clinic, these patients were not subjected to any further investigation in this study.

Patients with atrial fibrillation were divided into two groups with low or high risk for cardioembolic events. A patient with atrial fibrillation at low risk was defined one who had none of the following: age more than 60 years, previous cerebrovascular event, dilated cardiomyopathia, mitral stenosis, marked mitral insufficiency, or marked enlargement of the left atrium. All other patients with atrial fibrillation were defined as high risk. High risk was considered a legitimate reason for continuous anticoagulant treatment as long as the risk for bleeding complications was considered low. Low risk patients were invited for a medical examination, and discontinuation of anticoagulant treatment was considered, with a possible switch to other medications.

Patients with recurrent venous thromboses (three or more) without known risk factors were offered a medical examination and the presence of coagulopathy was examined. Anticoagulant treatment of these patients was continued if there were no contraindications.

All other legitimate indications were considered as relative indications, and they were weighed against the risk of bleeding complications. Risk factors and possible contraindications were: documented serious bleeding complications, as well as factors increasing risk of bleeding such as problems with compliance, fluctuating INR, balance problem with documented slip or fall accidents, dementia, liver failure and high age (>85 years of age).

Patients with unclear indication or with known risk factors were invited for further investigation. The indication for treatment was re-evaluated after laboratory tests and medical examination was performed. If no valid indication was found, or if the risk was found to be too high, anticoagulant treatment was discontinued or replaced with other appropriate medication whenever possible.

INR was determined with STA^® ^– SPA 50 kit (Diagnostica Stago, Asnieres-sur-Seine, France) on a Sysmex^® ^CA-6000 automatic coagulation instrument (Sysmex Corporation, Kobe, Japan). In mid-November INR values for the subjects were recorded. The first INR value dated on or after October 15, 2002 was recorded. In absence of such a value, the first INR value before this date was used. All values, except one, were determined within two months before this date.

Demographic data was collected from the SCB, Statistics Sweden website, , accessed January 6, 2005.

Information about warfarin use in Sweden was retrieved from the database PharmX supplied by Läkemedelsinformation AB.

Data was collected using a data sheet produced with SPSS Data Entry Builder 3.0, and SPSS™ version 12.0 (SPSS inc, Chicago, IL, USA) was used for statistical analysis. For comparison between groups, one-way ANOVA with Bonferoni post-hoc testing was used. A p-value <0.05 was considered statistically significant. All statistical tests and corresponding p-values were two-sided.

Ethical permission was granted by the local ethical committee of Umeå University.

## Results

We found 900 patients on continuous oral anticoagulant medication at the Oral Anticoagulant Clinic. On September 30, 2002 the total number of patients, both on short and long term treatment, registered in the outpatient clinic was 998. In addition, there were 57 patients monitored by general practitioners. On November 1, 2002, the population in the uptake area was 138 240 persons. Therefore, the total prevalence of oral anticoagulant treatment in the district was approximately 0.76%.

In the study by Jansson et al 1987–1990, the two most common indications for anticoagulant treatment were valvular heart disease (including mechanical valve prosthesis) and arterial thromboembolism [5]. In our study the most common indication for treatment was atrial fibrillation followed by mechanical heart valve prosthesis. Arterial thromboembolism is no longer one of the main indications (Table 1).

**Table 1:** Indications for warfarin treatment in 1987, 1990 (both continuous and temporary) and 2002 (continuous) at the Department of Internal Medicine and primary health care units in the Umeå district. Data extracted from Jansson et al. [5]. Only one indication was recorded. Hierarchical order 1987 and 1990: pulmonary embolism, deep venous thrombosis, ischaemic cerebrovascular disease, peripheral thromboembolism, valvular heart disease, atrial fibrillation and miscellaneous. Hierarchical order 2002: valvular heart disease, atrial fibrillation, arterial thromboembolism, venous thromboembolism and miscellaneous. When a patient had both arterial and venous events, the most recent event decided the group. Patients with missing records are not included in the table.

**Indications**	**1987**	**1990**	**2002**
	n (%)	n (%)	n (%)
Valvular heart disease	103 (42)	119 (41)	314 (37)
Arterial thromboembolism	70 (29)	82 (28)	11 (1)
Atrial fibrillation	18 (7)	21 (7)	339 (40)
Venous thromboembolism	47 (19)	52 (18)	60 (7)
Miscellaneous	5 (2)	16 (6)	120 (14)
**Total**	**243 (100)**	**290 (100)**	**845 (100)**

Mechanical heart valve prosthesis was present in 263 patients. Patient records of these patients were not examined, leaving 637 records to be found. We managed to find 582 patient records. Some indications and the mean target interval for these patients are presented in Table 2. Patient records of 55 subjects could not be retrieved. For these subjects some information was found through the warfarin dosage system, treatment indications of there are presented in Table 3. After evaluation of indications and contraindications, treatment was discontinued in 90 of the 582 patients due to dubious reasons for treatment or too high risk. Basic facts on these patients are presented in Table 4. Of these 90, the treatment of 23 patients was discontinued before the patient was invited for examination and by a physician who was not involved in this study (Table 5). All discontinuations, except one case that had atrial fibrillation, were in accordance with the method of re-evaluation. In four cases without a valid indication the medication was continued either because the patient or the patient's physician strongly opposed the discontinuation of warfarin treatment.

**Table 2:** Indications for oral anticoagulant treatment, age of patient, duration of treatment, mean target and, actual INR October 15, 2002. For mean target and INR, 10^th ^and 90^th ^percentiles are presented. All groups are compared with the Mechanical heart valve prosthesis group.

**Indication**	**n *(%)***	**Age (years)**	**Duration of treatment (years)**	**Mean target *(10–90%)***	**INR mean *(10–90%)***
Mechanical heart valve prosthesis	263 *(28.4)*	68.3	7.43	2.59*(2.55–2.85)*	2.68*(2.10–3.40)*
Atrial fibrillation	399*(44.3)*	75.4 ^a^	3.92 ^a^	2.51 *(2.40–2.55) *^a^	2.54*(1.90–3.20) *^c^
Other	183*(20.3)*	65.3 ^n.s.^	5.20 ^a^	2.50 *(2.25–2.55) *^a^	2.50*(1.90–3.20) *^b^
Record missing ^d^	55 *(6.9)*	70.9 ^n.s.^	4.67 ^a^	2.50*(2.15–2.55) *^a^	2.45 *(1.83–3.10) *^n.s.^
All indications	900 *(100)*	71.0	5.22	2.53 *(2.40–2.55)*	2.57 *(1.90–3.20)*

^a^p < 0.001, ^b^p < 0.01, ^c^p < 0.05, ^n.s^nonsignificant ^d^cf. Table 3

**Table 3:** Treatment indications for patients with missing patient record, data retrieved from warfarin dosage database

**Indication**	**n**	**%**
Atrial fibrillation	30	55
Thrombosis NUD	11	20
Valvular disease NUD	5	9
Cerebrovascular disease	3	5
Biological heart valve prosthesis	3	5
Myocardial infarction	2	4
Cardiomyopathia	1	2
Total	55	100

**Table 4:** Basic facts on patients whose treatment was continued vs. those whose treatment was discontinued.

	**Continued (n = 492)**			**Discontinued (n = 90)**			
	Mean (SD)	Min	Max	Mean (SD)	Min	Max	p
Age on October 15, 2002 (years)	72.4 (11.0)	20.4	94.7	71.2 (15.1)	20.6	92.6	0.370
Age at start of treatment (years)	68.2 (11.8)	17.2	89.1	66.1 (16.1)	13.7	89.7	0.149
Duration of treatment (years)	4.2 (3.6)	0.0	23.5	5.1 (4.5)	0.1	19.0	0.044
Mean target (INR)	2.5 (0.1)	1.7	3.2	2.5 (0.1)	1.7	3.2	0.249

**Table 5:** Reasons for discontinuation of oral anticoagulant treatment.

**Reason**	**n**	**%**
Lack of indication	46	51
Too high risk	21	23
Discontinued by physician outside the study	23	26
Total	90	100

Of all INR values recorded, using a cross-section method, 11.4% were below 2.0 and 15.2% above 3.0 in all long-term treated patients. For the subgroups continuously treated patients with mechanical valve prosthesis, atrial fibrillation, other indications and those with record missing the percentages were 7.0 and 22.7, 12.5 and 12.5, 13.7 and 12.0, and 16.1 and 11.3, respectively. Using a cumulative method for the period from January 1, to December 31, 2002, the percentages of patients in the total group, including short-term treatments that were outside the individual target intervals were higher, as well as the percentages outside INR 2.0–3.0 (Tables 6 and 7).

**Table 6:** Number of INR samples during 2002 under, within and over the patient's personal target interval

**2002**	**Under personal interval n (%)**	**Within personal interval n (%)**	**Over personal interval n (%)**
January-March	1274 (20.3)	3863 (61.7)	1125 (18.0)
April-July	1257 (20.7)	3791 (62.3)	1036 (17.0)
August-September	1299 (21.0)	3894 (62.8)	1005 (16.2)
October-December	1273 (20.5)	397 (63.8)	977 (15.7)
Total	5103 (20.6)	15521 (62.7)	4143 (16.7)

**Table 7:** Number of INR samples during 2002 <2.0, 2.0–3.0 and <3.0

**2002**	**INR <2.0 n (%)**	**INR 2.0–3.0 n (%)**	**INR >3.0 n (%)**
January-March	1032 (16.5)	4093 (65.4)	1137 (18.2)
April-July	1026 (16.9)	4040 (66.4)	1018 (16.7)
August-September	1033 (16.7)	4185 (67.5)	980 (15.8)
October-December	1047 (16.8)	4232 (68.0)	944 (15.2)
Total	4138 (16.7)	16550 (67.2)	4079 (16.5)

## Discussion

The total prevalence of 0.76% oral anticoagulant treatment in the reception area of the oral anticoagulant outpatient clinic was slightly lower than that reported, around 0.8% reported in Sweden in general [1] and in Denmark [2]. The population in the uptake area is young as compared with the rest of Sweden, 33% of the population was under 25 years of age, and 67% of the population was less than 50 years of age, as compared with 30% and 63% respectively, in the total Swedish population. We have not found any studies on continuous oral anticoagulant treatment to compare with our results.

A high number of patients with dubious indications and/or unacceptably high risk for treatment were found. The reason for this may be the fear of discontinuing anticoagulant medication. Those patients or their physicians may prefer the risk of a bleeding complication to the risk for a thromboembolic event. In addition, valid indications for oral anticoagulant treatment have changed, and the significance of coagulopathies may have been under- or overestimated in the past. This may also be the case for other indications. It takes time for new information to be implemented in everyday medical practice. In some cases, the patient or physician prefer to continue treatment despite lack of indication; this reflects some of the difficulties in routine oral anticoagulant monitoring.

A possible explanation for the high number of patients on questionable oral anticoagulant treatment may be that yearly re-evaluation is not always done due to lack of resources. Sometimes it may not be obvious who is in charge of the patient's oral anticoagulant treatment. In many cases the re-evaluation is made by the patient's primary health care doctor who doesn't always have access to the reasons for initiating the treatment or information about possible risk factors.

The change in indications as compared with the study done in 1987–1990 is striking (Table 1). Arterial thrombosis is no longer one of the main indications for oral anticoagulant therapy. A series of clinical trials that began in the mid-1980s provided substantial evidence for the effectiveness of warfarin in prevention of stroke in patients with atrial fibrillation [6-11]. The change may reflect the increasing awareness of this fact.

The most common target treatment range in this study was INR 2.1–3.0. After the completion of the study the target range had been changed to 2.0–3.0 in most of these cases. In a study by Samsa et al, 45.0–66.8% of the INR values in atrial fibrillation patients were outside the interval 2.0–3.0 [12]. In our study only 33.2% of all INR values during 2002 were outside this interval. In 1987 and 1990 Jansson et al found that less than 20% of the INR values at the oral anticoagulant clinic at Umeå University Hospital were outside the desired treatment interval 2.1–4.2 during that time period [5]. That was a much broader interval than we use nowadays. They also excluded patients whose observation time was too short, i.e., less than five INR values. We did not exclude any patients in our study due to short observation time. We also had several patients with actual target intervals different from the most common target interval 2.1–3.0. We therefore believe that the treatment quality in our study was at least as good as it was in the study by Jansson et al [5]. In that study PT ratio was used, but comparison is possible because our laboratory was responsible for all the analyses, using the same method principle (Owren), with the same reagents thus enabling us to calculate the INR values properly.

There has been consensus on the target range 2.0–3.0 in atrial fibrillation and also in most of the other indications [13,14]. Some patients, especially those with mechanical heart valve prostheses, have a higher target INR. This was seen especially among those whose treatment started a long time ago (data not shown). Some elderly people have lower target treatment range than INR 2.1–3.0. There is still a debate about the benefits and disadvantages of low-dose warfarin treatment after deep venous thrombosis [15-17]. In some cases a lowering of the treatment range might be an optimal alternative. In others it may be just an excuse for not discontinuing oral anticoagulant treatment. More studies are needed on this issue.

This study had some limitations: The prevalence of anticoagulant treatment may have been slightly underestimated since a few patients are treated through primary health care centres. However these patients are very few (n = 57). Complete patient records could not be found for 55 patients (8.6%) in the study, not counting those with mechanical heart valve prostheses. However, treatment indications of these patients were similar to those with records found. It can not be excluded that some bias of the results may be caused by the patients with missing records. The number of missing records will be lower when computerised medical records are introduced. However, in studies based on medical records, drop out rates higher than we found are common. The difficulty of retrieving medical records in a medical setting has been described [18]. In that study up to 30% of the patient records were unavailable at the time of the medical consultation. Some thromboembolic events in the past may have escaped our attention because they had been treated at another hospital.

There is a problem with the oldest of the old: The risk of thromboembolic events rises with age. So do the problems with maintaining the treatment within the target interval, and the risk of bleeding complications [19,20]. Attempts to prospectively assess risk factors in warfarin treatment have been made [21]. In medical practice, risk assessment is often subjective in the absence of accurate, objective, easy-to-use protocols. The balance between the risk for a thromboembolic event and the risk for a bleeding complication in this group is still unclear and further studies need to be done on this issue.

Thrombin inhibitors are a new group of drugs on the market. The fear of liver complications when using these drugs may prevent the wider usage of these drugs for many years, leaving anti vitamin K drugs an important option for secondary thrombosis prevention [22].

## Conclusion

In patients on oral anticoagulant therapy, re-evaluation of indications and risks resulted in a substantial number of treatment withdrawals. Treatment should be re-considered once a year, due to the rapid development of knowledge in the field of thrombosis risk factors. The indications for oral anticoagulant treatment have changed: Atrial fibrillation is now the most common indication while arterial thrombosis is no longer a common reason.

## Competing interests

The author(s) declare that they have no competing interests.

## Authors' contributions

KE, JH, BC and TS designed the study. KE examined the patient records and the patients. KE and TS made the clinical decisions on the patients. KE and JH analysed the data. KE, JH, BC and TS wrote the paper.
